# Resilient Aging: Psychological Well-Being and Social Well-Being as Targets for the Promotion of Healthy Aging

**DOI:** 10.1177/23337214211002951

**Published:** 2021-03-23

**Authors:** Eric S. Kim, Rifky Tkatch, David Martin, Stephanie MacLeod, Lewis Sandy, Charlotte Yeh

**Affiliations:** 1 University of British Columbia, Vancouver, Canada; 2 Optum, Southfield, MI, USA; 3 UnitedHealth Group, Minnetonka, MN, USA; 4 Optum, Ann Arbor, MI, USA; 5 AARP Services, Inc., Washington, DC, USA

**Keywords:** psychological well-being, social well-being, resilience, healthy aging, older adults

## Abstract

Population aging is one of the most important social trends of the 21st century and in the United States, the number of people aged ≥65 is projected to increase by nearly 50% in the next 15 years. Most biomedical and public health efforts have focused on reducing harmful risk factors when targeting chronic disease—an approach that has contributed greatly to prevention and treatment programs. However, evidence suggests that the number of years lost to disability is increasing and historic gains we have made in life expectancy are eroding, and even reversing in some groups. As our society ages and grapples with these issues, expanding the focus to include resilience, as well as psychosocial assets in our prevention and treatment programs might help inform the multidisciplinary response effort we need. Here we synthesize research evaluating associations between different dimensions of psychological well-being (e.g., purpose in life, optimism, life satisfaction) and social well-being (e.g., structural, functional, quality) with chronic conditions. We also evaluate evidence around three biopsychosocial pathways hypothesized to underlie these associations. These factors are meaningful, measurable, and potentially modifiable; thus, further pursuing this line of inquiry might unveil innovative paths to enhancing the health of our rapidly aging society.

In the United States the number of adults aged ≥65 is projected to increase by nearly 50% in the next 15 years (Colby & Ortman, 2014; Suzman et al., 2015). This rapid change in demographics is occurring in several nations throughout the world and for this reason and others, population aging is considered one of the most important social trends of the 21st century. Although average life expectancy has increased in the United States, so has the number of years lost to disability, and historic gains we have made in life expectancy over the last several decades are beginning to erode or even reverse in some groups (Murphy et al., 2018; Salomon et al., 2012). Further, US health-care spending, which reached $3.2 trillion in 2015, is expected to increase at an average rate of 5.5% per year over the next decade, an increase attributable partly to the rising prevalence and burden of chronic diseases. The burden of chronic disease rises with age and most psychological, biomedical, and public health efforts have focused on reducing harmful risk factors when targeting chronic disease—an approach that has contributed greatly to prevention and treatment programs. However, as our society ages and grapples with these issues, expanding the focus to include resilience, as well as psychological and social assets in our prevention and treatment programs might help inform the comprehensive and multidisciplinary response efforts our society needs.

Mounting research suggests that different dimensions of psychological well-being—which includes positive thoughts and feelings that people use to evaluate their lives favorably (e.g., a sense of purpose in life, optimism, and life satisfaction)—are uniquely associated with reduced risk of incident disease and premature mortality (Kim et al., 2019a; Kubzansky et al., 2018; Ryff, 2014; Steptoe, 2019; VanderWeele, 2017)—even after controlling for indicators of psychological distress (e.g., depression). Additionally, a broad body of work suggests that positive functioning in different dimensions of social relationships (e.g., structural, functional, quality) are also strongly associated with enhanced health outcomes (Berkman & Krishna, 2014; Hawkley & Cacioppo, 2010; Holt-Lunstad, 2018). These psychosocial assets are often included in the conceptualization of seminal gerontological and geriatric models that have emerged to characterize the antecedents, processes, and outcomes that foster people’s ability to age well in the face of accumulating adversities of older adulthood, including theoretical and empirical work on: “successful aging,” “optimal aging,” “effective aging,” “resilient aging,” “thriving,” and “positive aging” (Aldwin & Igarashi, 2015; Depp & Jeste, 2006; Fry & Keyes, 2010; Hochhalter et al., 2011; Lavretsky, 2014; Reich et al., 2010; Rowe & Kahn, 1987; Ryff et al., 1998; Ryff & Singer, 2009). However, echoing the seminal work of others (Friedman & Ryff, 2012a; Ryff & Singer, 2009), we agree that more work needs to evaluate the interplay between the biological, psychological, and social aspects of aging (biopsychosocial processes) when conceptualizing how people age well. In the spirit of continuous improvement, our hope is that by providing an overview of the state of the research evaluating psychosocial well-being and health research, and also highlighting research on potential mechanistic pathways through a biopsychosocial lens, it will spark further development of these seminal models. In addition to further developing conceptual models, our group—which consists of academics as well as specialists in healthcare: delivery, financing, operations, and benefit design—was interested focusing on how insights from these fields could translate into actionable next steps that could be deployed, at-scale, by large healthcare organizations.

To this end, we start by providing rationale for why we should consider resilience as a target for the promotion of healthy aging. We then discuss studies evaluating links between psychological and social well-being—factors that might contribute to resilience—with reduced risk of chronic disease incidence and premature mortality. Then, we evaluate evidence around three mechanistic pathways that might underlie associations between psychological well-being and social well-being with health outcomes. We end by discussing limitations of existing research and future research directions.

## Resilience as a Target for Healthy Aging

In the last ~100 years, our average life expectancies have increased by almost 30 years. However, due to structural lag our society’s core institutions (families, education system, workplaces, healthcare system, housing, design of neighborhoods, etc.) have not been adequately updated to serve the increasingly older age distribution that we are approaching (Rowe & Kahn, 2015). This structural lag means that our institutions, laws, and norms have failed to adapt to the reality of older adults who have limited opportunities, lack of support and preparation for the accumulating stressors that are faced including: social losses (e.g., death of parents, siblings, spouses, children, and friends), physical losses (e.g., declines in hearing, vision, functional abilities), and role-related losses (e.g., job-loss and loss of other community roles). Yet, in the face of these multifaceted and accumulating losses in older adulthood, some individuals, across the socioeconomic spectrum and across levels of health, remain resilient. Who are these individuals, and what social determinants of health and individual-level factors help them achieve this resilience? How are some able to adapt in the face of accumulating losses associated with older adulthood? If we identify the factors that foster resilience among the most resilient (across the socioeconomic and health spectrum) can such factors be cultivated in less resilient individuals through policy and intervention?

Resilience—a cluster of capacities, characteristics, resources, and processes related to the development and maintenance of healthy adaptation (Bonanno et al., 2015; Lavretsky, 2014; Pruchno et al., 2015)—is an attractive target for investigation and intervention, when compared to alternative definitions of healthy aging that often emphasize an objective health status. These conceptualizations do not easily include people with disabilities or impairments; on the other hand, enhancing resilience is an aim that all people can strive toward—regardless of their health status (Pruchno et al., 2015). Thus, even in the context of illness and disability, people can harness adaptive strategies to achieve a subjective sense of personal well-being and fulfillment (Young et al., 2009). Further, idealized models of healthy aging presuppose that there is one agreed upon set of criteria for older adults to strive for (Martinson & Berridge, 2015). However, focusing on resilience allows individuals to outline their own patterns of adaptive response which reflect their own values, heterogenous life course experiences (e.g., historical, cultural, and social contexts), and current circumstances (e.g., access to various assets, including: financial assets (savings, income, pensions), physical assets (e.g., infrastructure, shelter, transportation, sanitation), human assets (e.g., knowledge, skills, health, physical ability), social assets (e.g., networks, affiliation, reciprocity, trust) (Wild et al., 2013). No single factor exerts an overriding influence on resilience, but rather several antecedents exist (Bonanno et al., 2015). In this review, we focused on psychological and social factors that contribute to resilience because growing research suggests they are factors that are measurable, modifiable, and linked with mechanistic processes and outcomes that contribute to healthy aging. In the following section, we provide a brief overview of psychological and social well-being factors and simultaneously contribute to processes that enhance resilience, and reduce the risk of age-related chronic conditions.

## Psychological Well-Being, Social Well-Being, and Health

Psychological well-being has been defined in various ways, and although its exact content and contours are contested and evolve with new empirical research and theoretical models (Kashdan et al., 2008), two main perspectives characterize its essential features. First is the eudaimonic approach, which defines psychological well-being as a person’s ability to identify meaningful pursuits, and the act of striving toward them through virtuous activities in pursuit of achieving one’s ultimate potential (Ryff, 2014). Second, is the hedonic approach, which defines psychological well-being as a high frequency of positive affect, low frequency of negative affect, and the evaluation of life as satisfying (Diener et al., 1999). Other psychological constructs that fall under the broad umbrella of psychological well-being but do not fit neatly into either theoretical approach exist (e.g., optimism).

Accumulating research has documented associations between psychological well-being and reduced incidence of several age-related conditions (e.g., lung disease, cognitive function, cardiovascular disease) and slower decline in physical function (Kim et al., 2019a, 2019b; Kubzansky et al., 2018; Ryff, 2014; Steptoe, 2019; VanderWeele, 2017; VanderWeele et al., 2020a). For example, one study of 453 older adults from the Rush Memory and Aging Project (mean age = 84) who were followed over 6 years (and on their death, autopsied and diagnosed by neurologists) found that a higher sense of purpose in life was associated with lower odds of macroscopic infarcts (odds ratio = 0.54, 95% CI: 0.35–0.83), but not microinfarcts (Yu et al., 2015). Further, some studies have begun evaluating psychological well-being in relation to cognitive function and find that a sense of purpose in life and optimism are both associated with reduced risk of cognitive impairment and Alzheimer disease (Boyle et al., 2010; Gawronski et al., 2016; Oh et al., 2020). Finally, one study with 3,577 older adults examined the influence of purpose in life, resilience, optimism, internal locus of control, and social connections on health outcomes (Musich et al., 2020). This study found that those with high levels of any of these psychosocial factors had better physical functioning, better health status, and lower healthcare utilization.

Recent meta-analyses have also shown that several dimensions of psychological well-being are associated with reduced risk of mortality. In a recent meta-analysis of 90 prospective studies (pooled *n* = 1,259,949), higher psychological well-being was associated with small to moderate reduced risk of mortality (hazard ratio = 0.92; 95% CI = 0.91–0.93) (Martín-María et al., 2017). Another meta-analysis of 10 prospective studies (pooled *n* = 136,265) showed that a higher sense of purpose in life was associated with reduced risk of mortality (hazard ratio = 0.83; 95% CI = 0.75–0.91) (Cohen et al., 2016). The studies in these meta-analyses used prospective designs, had reasonable follow-up times, and adequately controlled for key confounders. Psychological factors alone are likely insufficient when considering healthy aging, thus we also turn to social relationships.

Social networks are often measured structurally (e.g., social contact frequency, living alone), functionally (e.g., perceptions of social support, perceived loneliness), and/or by its quality (e.g., marital quality, relationship strain) (Holt-Lunstad, 2018). Growing research has documented associations between poor social relationships and greater incidence of several age-related conditions. For example, a meta-analysis of 19 prospective studies observed that low social participation (RR = 1.41, 95% CI = 1.13–1.75), less frequent social contact (RR = 1.57, 95% CI = 1.32–1.85) and higher loneliness (RR = 1.58, 95% CI = 1.19–2.09) were associated with higher incidence of dementia. However, the meta-analysis did not observe a relationship between satisfaction with social network and dementia incidence, and also found inconsistent results when evaluating social network size (Lara et al., 2019). Additionally, lack of social resources has often been studied in the context of cardiovascular disease (CVD) and one meta-analysis of 19 studies (pooled *n* = 181,006) reviewed loneliness and social isolation as risk factors of coronary heart disease and stroke; the meta-analysis observed that low social resources was associated with a 29% increased risk of incident CHD (95% CI = 1.04–1.59) and 32% increased risk of stroke (95% CI = 1.04–1.68) (Valtorta et al., 2016).

Social relationships are also associated with reduced risk of mortality as illustrated in a recent meta-analysis of 70 studies (pooled *n* = 3,407,134), which observed that several objective and perceived dimensions of poor social relationships were associated with increased risk of mortality: social isolation (OR = 1.29, 95% CI = 1.06–1.56), loneliness (OR = 1.26, 95% CI = 1.04–1.53), and living alone (OR = 1.32, 95% CI = 1.14–1.53) (Holt-Lunstad et al., 2015). Another recent meta-analysis of 91 studies (pooled *n* = 400,000) observed that those with lower levels of social contact frequency had a 13% increased risk of mortality (95% CI = 1.09–1.17) (Shor & Roelfs, 2015). These studies provide suggestive evidence that psychological and social well-being are associated with reduced risk of chronic conditions, but research has yet to clearly identify potential underlying mechanisms.

## Mechanisms: Psychosocial/Stress-Buffering, Behavioral, and Biological Pathways

**Figure 1.**
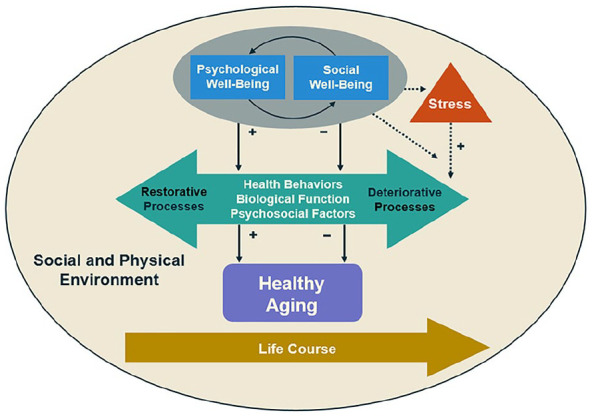
Relationship between psychological, and social, well-being with chronic conditions. This model illustrates the relationships linking psychological well-being and social well-being with chronic conditions via health behaviors, biological, function, and psychosocial factors. Additionally, psychological well-being and social well-being may reduce the likelihood of appraising stressors as stressful, but when stress is experienced it may buffer against the health-related impact of excessive stress. Further, we acknowledge that these relationships are all embedded within the life course and heavily influenced by both social and physical environmental factors. For the purpose of parsimony we present a unidirectional model. However, the exclusion of alternative paths is not intended to reflect hypotheses about their existence, nor imply that these relations are static. +Positive relationships. −Negative relationships.

In the face of accumulating adversities and stressors associated with aging (e.g., social, physical, role-related losses), psychological and social well-being might influence health through at least three biopsychosocial pathways: (1) the promotion of other psychosocial factors that buffer against the harmful effects of overwhelming stress, (2) effects through health behaviors, and (3) effects on biological processes. All three processes are hypothesized to reduce “wear and tear” on the body, which in turn reduces people’s vulnerability to age-associated chronic diseases. Figure 1, adapted from previous work (Kubzansky et al., 2018), illustrates each pathway and shows how psychological and social well-being might enhance the likelihood of restorative processes (e.g., healthy sleep) and decrease the likelihood of deteriorative processes (e.g., smoking) (Kubzansky et al., 2018). Additionally, psychological and social well-being might help people appraise stressors as less severe and also foster quicker recovery when stress is experienced (Figure 1). This model also acknowledges that these processes unfold over the life course and are shaped by our social (e.g., social determinants of health) and physical environment. In the following section, we highlight findings evaluating associations between psychological well-being, and social well-being, with each of these three pathways and emphasize processes particularly relevant to aging. Although most existing studies are cross-sectional, we highlight studies that use methodologically stronger designs (e.g., longitudinal data or experimental).

### Psychosocial Pathways and Stress Buffering

Although perceived stress typically declines with age,(Stone et al., 2017) it is important to identify factors that help buffer against it. Optimism is perhaps the facet of psychological well-being with the most research in relation to age-related chronic conditions. Thus, we discuss research on optimism to illustrate how various psychosocial mechanisms might mediate the association between psychological well-being and reduced risk of chronic conditions. Optimism provides a confidence about the future that might foster several psychological and cognitive mechanisms that enhance health including how a person: (1) self-regulates, (2) perceives daily stressors, (3) engages with one’s goals (from a motivational perspective), (4) copes with challenges, and (5) adjust goals if they become unattainable (Rasmussen et al., 2006). As a result, people with higher optimism might be less inclined to activate the stress-linked neurohormonal cascade (i.e., activating the sympathetic-adrenal medullary system and hypothalamic-pituitary-adrenocortical axis, or dampening of parasympathetic nervous system), or to engage in unhealthy coping behaviors that increase risk of age-related conditions.

Self-regulation involves responses in the cognitive, affective, and/or behavioral domains in any given situation and in the context of one’s larger goals (Vohs & Baumeister, 2011); these qualities provide the means with which to confront and adapt effectively to life’s challenges (Kubzansky et al., 2014). Optimism appears to alter processing and interpretation of daily stressors so they are experienced as less threatening (Peters et al., 2016); thus, optimism might enhance a person’s capacity to regulate emotions in response to daily stressors. Individuals react to similar stressors differently and some individuals experience substantially more negative affect relative to others (Almeida et al., 2011). This “emotional reactivity” reflects individual variation in likelihood to respond to stimuli with high negative affect or decreases in positive affect (Almeida et al., 2011; Mroczek et al., 2015). High emotional reactivity is associated with adverse health outcomes including higher inflammation and mortality risk (Mroczek et al., 2015; Sin et al., 2015). More adaptive emotion regulation strategies (e.g., changing how one views an emotion-eliciting event/cognitive reappraisal) versus maladaptive strategies (e.g., suppressing the expression of emotion) are associated with lower inflammation and more favorable cardiovascular health (Applebaum & Kubzansky, 2014). Thus, research suggests that optimism might lead to less emotional reactivity in response to stressful circumstances, and increased use of adaptive emotion regulation strategies (John & Gross, 2004) (e.g., persevering and coping by using problem-solving and planning strategies to manage the stressor (Carver et al., 2010; Nes & Segerstrom, 2006); however, when faced with uncontrollable stressors, shifting sights to other goals and using adaptive emotion-focused coping mechanisms, such as acceptance of the current situation (Aspinwall & Richter, 1999).

Several mechanisms help explain the associations observed in past research between social relationships and health, including: self-efficacy, the role of spouses, healthier coping styles and appraisals of situations, and the fostering of psychological well-being. Self-efficacy—belief in one’s ability to accomplish a task or succeed in specific situations—is one key hypothesized mechanism and research suggests that social relationships enhance self-efficacy (Berkman & Krishna, 2014), which in turn is associated with a range of healthier behaviors including more physical activity and smoking cessation (Berkman & Krishna, 2014). From a dyadic perspective, one study observed that among close partners, an individual in the couple felt more efficacious about changing his/her behavior if his/her partner was at a higher stage of readiness to change their health risk behavior (Franks et al., 2012). Subsequent research shows that improvement in an individual’s behavior (smoking cessation, physical activity, weight loss), predicts improvement in that same behavior in the spouse (Jackson et al., 2015).

Other evidence suggests that social relationships promote healthier coping styles and appraisals of situations. For example, those with high levels of perceived social support feel increased levels of security because they are less likely to appraise a stressful situation as threatening, thus blunting the likelihood of physiological dysregulation (Ditzen & Heinrichs, 2014). Further, social relationships might also work through enhanced likelihood of psychological well-being and decreased risk of psychological distress. The “brain opioid theory of attachment” suggests that positive social interactions generate endogenous opioids to reinforce them as rewarding experiences (Loseth et al., 2014), and conversely a meta-analysis of 51 studies demonstrates that several dimensions of social relationships help buffer against the risk of depression (Santini et al., 2015). The array of psychosocial resources that people with higher psychological and social well-being possess, might influence people’s health behaviors.

### Behavioral Pathways

Psychological well-being might enhance the likelihood that people engage in restorative health behaviors (e.g., use of preventive services, physical activity, healthy eating) while also decreasing the likelihood that people engage in harmful behaviors (e.g., smoking). Most studies in this domain use self-report data to assess both psychological well-being and health behaviors. Thus, common-method bias and reverse causality remains concerns. Thus, we highlight studies using longitudinal designs and objective measures where available. A recent meta-analysis evaluating the association between optimism and three health behaviors documented how more optimistic people (compared to less optimistic people) were modestly more likely to display greater physical activity (34 effect sizes; *n* = 90,845; *r* = 0.07) healthier diets (15 effect sizes; *n* = 47,931; *r* = 0.12), and less smoking (15 effect sizes; *n* = 15,053; *r* = 0.07) (Boehm et al., 2018).

Numerous other studies have documented associations between other dimensions of psychological well-being and healthier behaviors (e.g., greater physical activity, healthier diets, higher quality sleep, and less smoking) (Kubzansky et al., 2018). Some of the newer research in this area used more rigorous methods (e.g., longitudinal study designs, adjusting for relevant covariates, use of validated measures of exposure and outcomes). For example, in a large prospective study of 9,986 healthy British adults from the English Longitudinal Study of Aging who were followed over an average of 11 years (and assessed repeatedly up to six times), higher psychological well-being was associated with higher physical activity over follow-up after adjusting for several potential confounders, including psychological distress (Kim et al., 2017). Other studies have observed that people with higher psychological well-being are more likely to use preventive healthcare screenings (e.g., cholesterol tests and cancer screenings) and also make less use of emergency services—an indicator of health (Kim et al., 2014, 2015; Musich et al., 2017; Wilson et al., 2018).

Social relationships might promote health behaviors through a variety of mechanisms including: social support (e.g., instrumental, financial, informational, appraisal, emotional), social influence (e.g., constraining and enabling norms toward: health behaviors, help seeking, adherence), social engagement (e.g., physical and cognitive stimulation, maintenance of meaningful social roles), access to resources (e.g., economic opportunities, access to health care, housing, institutional contacts), and negative social interactions (e.g., demands, criticisms, perceived isolation) (Berkman & Krishna, 2014; Hawkley & Cacioppo, 2010; Holt-Lunstad, 2018). A recent systematic review found that evidence linking social isolation and loneliness with health behaviors is mixed (Leigh-Hunt et al., 2017). For example, a review evaluating loneliness in relation to smoking identified 25 studies, and only half reported an association between higher loneliness and smoking (Dyal & Valente, 2015). A recent review of 27 studies evaluating the association between social support and physical activity found that although the association between general social support and physical activity was inconsistent, specific forms of social support for physical activity from family (but not from friends) were associated with increased physical activity (Smith et al., 2017). The review also observed that loneliness had mixed findings in relation to physical activity. Another review of 28 studies evaluated social isolation and loneliness in relation to malnutrition in older adults, and found no association (van der Pols-Vijlbrief et al., 2014).

Another line of research used social network analyses on large epidemiological cohort studies (e.g., Framingham Heart Study and the National Longitudinal Study of Adolescent Health) to demonstrate that at least 15 different health-related behaviors can be “socially transmitted” (e.g., obesity, smoking, sleep quality) (Christakis & Fowler, 2013). One illustrative example focused on smoking and tracked the interconnected social network data of 12,067 participants in the Framingham Heart Study over a 32-year period. The researchers observed that a person’s likelihood of smoking was 61% higher if the contact was only one-degree of separation away, 29% higher if the contact was two-degrees of separation away, and 11% higher if the contact was three-degrees of separation away (Christakis & Fowler, 2008). Further, smoking cessation occurred in synchrony among clusters of people, suggesting that a strong social force influences certain health behaviors. As people with a higher sense of psychological and social well-being engage in healthier behaviors, it might translate into healthier biological function.

### Biological Pathways

Growing research links different dimensions of psychological well-being with key biological processes and markers of aging and age-related chronic conditions including: physiological function (e.g., cardiovascular function, lung function, glucose metabolism, body composition), physical function (e.g., strength, balance, dexterity, locomotion), endocrine function (Hypothalamic–Pituitary–Adrenal axis (HPA-axis), sex hormones). A limitation of this work is that most studies are cross-sectional, although a growing number of studies use longitudinal and experimental designs. However, one advantage of studies in this area is that biological outcomes are objectively assessed—reducing concerns about self-report and common-method bias.

When turning to physiological function and cardiovascular health specifically, we consider sense of purpose in life’s association with cardiovascular biomarkers as an illustrative example. A higher sense of purpose has been associated with enhanced glucose regulation (Boylan et al., 2017; Hafez et al., 2018; Rasmussen et al., 2013). When considering inflammation, however, sense of purpose does not appear to have direct associations (Cole et al., 2015; Friedman et al., 2007; Friedman & Ryff, 2012b; Morozink et al., 2010; Steptoe & Fancourt, 2019), but instead act as an effect modifier that “dampens” the effect of factors that increase inflammation. For example, among people with either chronic health conditions or low socioeconomic status, people who also reported high purpose in life, exhibited lower levels of inflammation. Studies evaluating other cardiovascular markers including: atherosclerosis, carotid intima thickness, or coronary artery calcification observed no associations with sense of purpose (Low et al., 2011; Shahabi et al., 2016; Sloan et al., 2017).

Mounting research has also linked different dimensions of social relationships with key biological processes and markers. One recent and illustrative study evaluated social relationships in relation to physiological dysregulation in four nationally representative epidemiologic studies. Among the two cohort studies focusing on older adults, higher social integration was associated with lower odds of inflammation in one of the cohorts (OR = 0.89, 95% CI = 0.81–0.98) and hypertension in both of the cohorts (OR = 0.87, 95% CI = 0.78–0.89 and (OR = 0.46, 95% CI = 0.25–0.85) (Yang et al., 2016); however, the associations between social integration and abdominal obesity, or overall obesity, were null. A meta-analysis also evaluated inflammation in 41 studies (pooled *n* = 73,037) and found that both social support and social integration were associated with lower levels of inflammation (*Zr* = −0.073) (Uchino et al., 2018).

## Future Research Directions: Basic Research

As we evaluate gaps in the existing literature, we see several exciting future directions that have the potential to provide greater insight into how psychological and social well-being might influence healthy aging, and also whether such insights could potentially translate into scalable interventions.

### Outcome-Wide Methodology

Various research findings demonstrate conflicting results. The underlying reasons for conflicting past results remain unclear but might be due to truly diverging observations or methodological differences. For instance, these may include (1) different study designs, (2) different populations, (3) controlling for different numbers and types of confounders, (4) key underlying moderators that have not been addressed, (5) different effects on different outcomes, and (6) the use of a wide array of different measures to assess the exposure. One way to help address whether conflicting results are due to methodological factors is to use large epidemiologic datasets that have varying measures of exposures (e.g., numerous measures of social relationships) and then standardize all the study design factors (e.g., study design, population, covariates) via an outcome-wide approach (VanderWeele et al., 2020b), to assess which dimensions of social relationships are truly the “active ingredients.”

### Measurement

Researchers have noted that some existing assessments of psychological well-being, such as optimism, may not be sensitive enough to detect changes in the construct as they were originally created to measure traits instead of states. Thus, new measures have been developed to address this issue (Millstein et al., 2019), and future research should harness these measures and compare them to more traditional options when considering health outcomes. Additionally, emerging theoretical work has led to developments in the conceptualization of some dimensions of psychosocial well-being. For example, a tripartite model of meaning in life has recently emerged, which consists of three subcomponents: (1) *purpose* in life; (2) coherence/comprehension; and (3) significance/mattering (George & Park, 2016; Martela & Steger, 2016). Current evidence evaluating chronic disease risk has been strongest for purpose in life, but this is potentially a byproduct of the fact that it is typically the only facet of the tripartite model assessed in studies. Thus, to advance knowledge and identify the best potential targets for intervention, studies should also consider assessing other facets of psychosocial well-being as they emerge.

## Future Research Directions: Applied Research

### Interventions Delivered via Healthcare Systems

When considering potential interventions that could be disseminated at-scale via the healthcare system, several future directors are of interest. Several studies evaluating psychological well-being interventions have begun considering issues related to effective translation and found that adjusting the dosage and variety of the active ingredients can enhance the likelihood that strategies for improving psychological well-being will be adopted, implemented, and maintained (Layous & Lyubomirsky, 2014). Additional, work has begun to identify the most critical components of interventions (e.g., duration, intensity, content) (Celano et al., 2018), using multiphase optimization strategy and sequential multiple assignment randomized trials—which allow researchers to test and identify the best performing intervention components simultaneously and efficiently (Brown et al., 2017).

Several recent meta-analyses of randomized controlled trials suggest that certain interventions can improve psychological well-being (Bolier et al., 2013; Malouff & Schutte, 2016; Weiss et al., 2016). Existing interventions have used a variety of methods including writing about positive events, expressing gratitude, practicing prosocial behavior, and individual or group therapy (based on cognitive behavioral therapy (CBT) principles). A limited number of studies have begun targeting psychological well-being as a method of improving health-related outcomes. As an example, recent research conducted by included smaller interventions to improve psychological well-being, resilience, and social connections using web-based mindfulness modules tailored for older adults and animatronic pets (Hudson et al., 2020a, 2020b; Tkatch et al., 2017, 2020). In another area, researchers evaluated whether a combination of positive affect and self-affirmation interventions influence physical activity and medication adherence among patient groups. Researchers conducted three randomized trials targeting at-risk patient groups on a medical regimen, including people with asthma, hypertension, or heart disease. All study participants received patient education about their particular condition, but participants in the intervention group were taught and encouraged to use strategies that induced positive affect and self-affirmation (e.g., positive moments, core values). As an illustrative example, in one study of patients who recently had a cardiovascular procedure, everyone’s within-person change in physical activity was tracked for 12 months. People in the treatment group (who received positive affect and self-affirmation interventions) were 1.7× more likely to achieve a standardized physical activity goal when compared to participants in the control group (Peterson et al., 2012).

In the space of social relationship interventions, researchers have created an array of methods. In their review of the loneliness intervention literature Cacioppo et al. (2015) grouped interventions into four primary categories; (1) increasing social contact (2) improving social support (3) enhancing social skills (4) addressing maladaptive social cognition and found that the most effective interventions addressed maladaptive social cognition via cognitive behavioral therapy (Cacioppo et al., 2015). Another review of 38 social isolation interventions identified three elements that most enhance the efficacy of interventions including: (1) adaptability, (2) a community development approach, and (3) productive engagement (Gardiner et al., 2018). When considering the dissemination of social relationship interventions at a population level, Holt-Lunstad (2018) urges the use of a more preventive approach, as well as a multi-level systems approach that targets causal mechanisms across each level (e.g., individual-, relationship- or family-, community-, and societal-levels) (Holt-Lunstad, 2018).

When considering all these factors, one question that repeatedly emerges is the potential exchangeability of psychosocial well-being dimensions. For example, are optimism, positive affect, and purpose in life exchangeable when considered in relation to health outcomes? If a person is low on any of these dimensions, can other dimensions of psychological well-being compensate?

### Policy-Level Interventions

Several prominent intergovernmental organizations (e.g., Organization for Economic Co-operation and Development, World Health Organization, United Nations) are urging countries to use well-being indicators (e.g., life satisfaction), in addition to traditional economic indicators (e.g., gross domestic product), when making important policy decisions (Stiglitz et al., 2018; The Global Council for Happiness and Wellbeing, 2019; World Health Organization [WHO], 2013). A growing number of countries have already adopted well-being measures as metrics and decision-making tools to guide policy decisions, and several others are on the horizon (Stiglitz et al., 2018; The Global Council for Happiness and Wellbeing, 2019; WHO, 2013). Emerging evidence suggests that life satisfaction can be enhanced through a variety of population-level intervention programs and policies. As nations pause and re-evaluate priorities in light of the widespread change that COVID-19 and its downstream effects have caused, our policymakers have a rare opportunity to pursue factors like satisfaction and several other dimensions of psychosocial well-being as a policy aim.

## Conclusion

As our population ages a comprehensive, multidisciplinary, and multi-level effort is needed across disciplines and sectors to enhance the health and well-being trajectory of those moving into the ranks of our aging society. Resilience and psychosocial well-being might be novel intervention targets and key pieces needed in this comprehensive response. Although many outstanding questions remain, evidence across multiple disciplines is converging to suggest that identifying, understanding, and intervening upon psychosocial well-being (instead of focusing exclusively on reducing risk factors) might provide innovative paths for enhancing healthy aging at the population level. Thus, a focus on how psychosocial well-being might be altered at the population-level and individual-level (e.g., targeted interventions delivered through healthcare systems) might open new ways of simultaneously enhancing the quality of life, and physical health, of those moving into the ranks of our aging society.
